# Hub genes, diagnostic model, and predicted drugs related to ferroptosis in chronic rhinosinusitis with nasal polyps

**DOI:** 10.1097/MD.0000000000040624

**Published:** 2024-11-29

**Authors:** Qian Guo, Dong Dong, Xinjie Qiao, Shuman Huang, Yulin Zhao

**Affiliations:** a Department of Rhinology, The First Affiliated Hospital of Zhengzhou University, Zhengzhou, China; b Key Laboratory of Otolaryngology Diseases, Henan Provincial Health Commission, Changsha, China.

**Keywords:** chronic rhinosinusitis with nasal polyps, diagnostic, drug, ferroptosis, hub gene, immune

## Abstract

Significant progress has been made in the pathogenesis of chronic rhinosinusitis (CRS). However, the relationship between chronic rhinosinusitis with nasal polyps (CRSwNP) and ferroptosis, as well as its underlying molecular mechanism, remains unclear. This study aimed to investigate the correlation between CRSwNP and ferroptosis and identify key gene associated with ferroptosis that could impact the diagnosis and treatment of CRS. To achieve this, gene expression profiles containing CRSwNP and CRSsNP samples were obtained from the GEO database. In addition, from the FerrDb V2 database, we acquired 2 sets of genes that are connected with ferroptosis, giving us a combined number of 260 genes associated with this particular biological process. Differential analysis and weighted gene co-expression network analysis (WGCNA) were performed on nasal tissue samples from GSE36830, leading to the identification of 1 key gene related to ferroptosis and CRS. Using stepwise regression and logistic regression analysis, we constructed a diagnostic model for CRS using ALOX15. The AUC value demonstrates that the model exhibits a strong diagnostic performance. Furthermore, the connection between immune cell infiltration in the samples and hub gene was explored, suggesting the potential significance of the hub gene in the immune response to CRS. Finally, Five drugs targeting a central gene were identified from the DrugBank database, and a few of them have exhibited efficacy in the treatment of CRS or associated ailments. In conclusion, this model holds potential for supporting the diagnosis of CRS patients, while the central gene identified may contribute to a better understanding of CRS development and drug treatment.

## 1. Introduction

Chronic rhinosinusitis (CRS) refers to the enduring inflammation occurring in the nasal cavity and the paranasal sinuses, significantly affecting patients’ quality of life (QoL).^[1]^ This condition is characterized by enduring symptoms, including nasal congestion, facial pain, headaches, and impaired olfactory function.^[2]^ The impact of CRS on quality of life extends beyond physical symptoms. It often leads to sleep disturbances, fatigue, emotional distress, and decreased social participation, all of which can markedly impair daily functioning.^[3,4]^ The clinical course of chronic sinusitis exhibits considerable variability between children and adults, resulting in differing impacts on quality of life. In children, chronic sinusitis is frequently linked to underlying allergic or immune factors, as well as structural abnormalities such as adenoidal hypertrophy.^[4]^ In adults, CRS is commonly associated with environmental exposures, recurrent infections, or systemic diseases, which can lead to significant complications, including increased susceptibility to long-term inflammation, nasal polyposis, and a heightened risk of surgical intervention.^[5,6]^ Research indicates that the primary detriment to the health of children with chronic rhinitis and sinusitis is linked to the emotional distress, pain, and discomfort experienced by their parents, along with the family’s overall perceptions of health.^[7]^ Nowadays, biologics are available to treat chronic sinusitis with nasal polyps.^[8]^ However, it is necessary to gain a deeper understanding of the pathophysiology of chronic rhinosinusitis with nasal polyps (CRSwNP) to effectively tailor treatments to the specific disease endotype. Therefore, the molecular mechanism of CRSwNP remains to be further elucidated.^[9]^ Ferroptosis is an iron-dependent, oxidatively regulated form of cell death that occurs due to the buildup of lipid peroxides and reactive oxygen species.^[10]^ The primary mechanism underlying ferroptosis involves the elevated presence of unsaturated lipids, which undergo catalysis by either ester oxygenase or divalent iron within the cellular membrane.^[11]^ This catalysis leads to lipid peroxidation, which in turn induces cell death. The process of ferroptosis is complex and involves a variety of biomolecules and metabolites.^[12–14]^ In the past few years, a growing body of research has validated that ferroptosis is an emerging variant of cellular demise connected to inflammatory processes.^[15,16]^ Furthermore, abnormal iron metabolism is associated with elevated inflammatory markers in nasal and sinus tissue in patients with CRSwNP.^[17]^ In short, the relationship between ferroptosis and CRSwNP cannot be ignored. Relationship. Although significant progress has been made in the field of CRSwNPs, the understanding of ferroptosis and its molecular mechanisms in CRSwNPs is still unclear. Therefore, we studied the relationship between CRSwNP and ferroptosis, with the aim of further elucidating its mechanism and exploring more promising targeted treatments.

**Figure 1.**
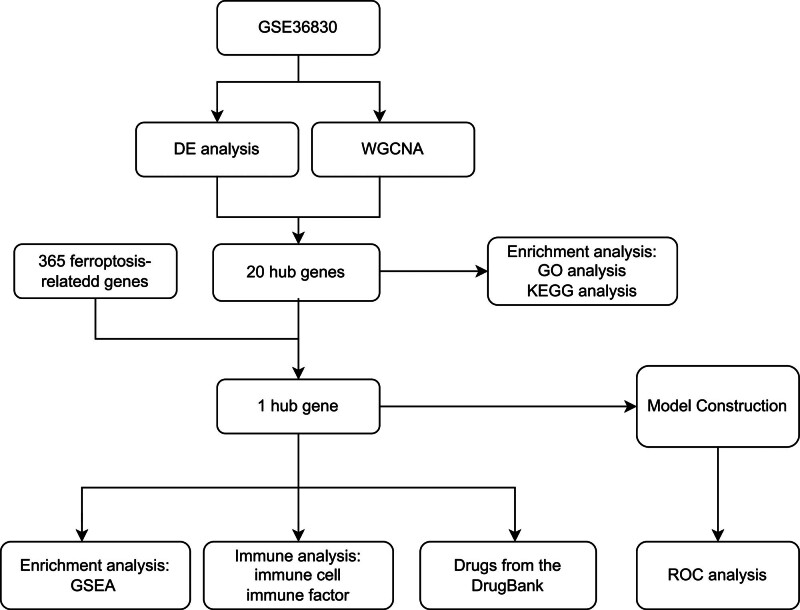
The workflow of the analyses.

Although significant progress has been made in the field of CRSwNPs, the understanding of ferroptosis and its molecular mechanisms in CRSwNPs is still unclear. Therefore, we studied the relationship between CRSwNP and ferroptosis, with the aim of further elucidating its mechanism and exploring more promising targeted treatments. This study utilized ample public resources and bioinformatics techniques to identify a hub gene that is associated with ferroptosis and CRSwNP. This was accomplished through performing differential analysis and WGCNA = weighted gene co-expression network analysis (WGCNA) on GSE36830 dataset. Additionally, Gene Set Enrichment Analysis (GSEA) analysis was conducted to delve deeper into the examination of biological processes and pathways. A diagnostic model for CRSwNP was constructed using stepwise regression and logistic regression analysis with 1 gene. The AUC value suggests the model displayed excellent diagnostic capabilities and has potential applications in the clinical diagnosis of CRSwNP. Sinus inflammation is a significant pathological feature of CRSwNP and is closely associated with the immune system. Hence, we extensively investigated the infiltration of the immune system in these specimens and explored the association between various immune cells and the pivotal genes. Moreover, we obtained 5 medications that specifically target core genes from the DrugBank database, which is of great significance for the drug treatment of CRSwNP. The workflow of this study is shown in Figure 1

## 2. Materials and methods

### 2.1. Patients and tissue samples

This study adhered to the principles outlined in the Declaration of Helsinki and received approval from the Life Sciences Ethical Review Committee at Zhengzhou University. We recruited 5 patients with CRSwNP, 5 patients with chronic rhinosinusitis without nasal polyps (CRSsNP), and 5 control subjects. Each participant provided written informed consent. The diagnosis of CRS was made according to the European Position Paper on Rhinosinusitis and Nasal Polyps (EPOS) 2012 guidelines. The control group comprised patients diagnosed with pituitary tumors, cerebrospinal fluid leaks, or maxillary sinus cysts. Patients with the following conditions were excluded from this study: allergic fungal sinusitis, autoimmune diseases, acute sinusitis, aspirin intolerance triad, cystic fibrosis, or primary ciliary dyskinesia. None of the patients received oral or nasal steroids, or any other medications (including antibiotics or antileukotriene drugs) within 1 month prior to surgery. The methods for sample collection have been outlined in previous studies. In summary, nasal polyp (NP) tissue was collected from CRSwNP patients, uncinate process mucosal tissue from CRSsNP patients, and mucosal tissue from control subjects during surgical procedures.

### 2.2. Data acquisition

The data regarding gene expression were acquired from the public database of NCBI Gene Expression Omnibus (GEO). Specifically, the GSE36830 dataset comprises annotated RNA expression data of superior turbinates using the GPL570 platform., which includes 12 CRSwNP and 6 CRSsNP sample controls.

### 2.3. Differential expression analysis

Differential expression analysis of CRSwNP and CRSsNP samples was performed using the “limma” package in R software. Genes with *P*_adj < .05 and abs(logFC) > .585 were considered DEGs. Heatmaps and volcano plots were generated to visualize the DEGs using the “pheatmap” and “ggplot2” packages.

### 2.4. Ferroptosis-related genes

The ferroptosis-related genes, including drive and suppressor genes, were obtained from the FerrDb V2 database.^[18]^

### 2.5. Weighted correlation network analysis (WGCNA)

In order to explore the co-expression patterns of genes and their relationships with phenotypes, we utilized the “WGCNA” package in R software^[19]^ to establish a gene co-expression network. After analyzing the cluster trees, we removed samples from the dataset that showed unusual traits. We retained the top 5000 genes characterized by a median absolute deviation (MAD) >1. Constructing a similarity matrix involved calculating the correlation coefficient between every pair of genes. In order to accomplish the establishment of a network that follows the scale-free property, a suitable soft threshold was chosen to transform the matrix of similarities into an adjacency matrix. Afterwards, the creation of topological overlap matrix (TOM) occurred, aiming to evaluate the average connectivity level of every gene. Using the dynamic tree cutting technique, genes exhibiting similar expression patterns were classified into distinct modules based on various parameters such as minModuleSize and mergeCutHeight within the blockwiseModules function. Each module was represented by a distinct color, while the genes in gray modules were those that did not belong to any specific module. To determine the gene expression profile of each module, we utilized a principal component known as the module eigengene (ME). To assess the correlation between the modules and phenotypes, we utilized the MEs. The module that exhibited a significant absolute value of the correlation coefficient was identified as the central module for further investigation. Module membership (MM) is defined as the correlation coefficient that quantifies the association between a gene’s expression value and the ME of a module. This measurement indicates the level of correlation between the gene and the particular module. Gene significance (GS) depicts the correlation coefficient between the expression value of a gene and a phenotype, exemplifying the connection between genes and phenotypes.

### 2.6. Identification of hub gene

To obtain hub gene linked to both ferroptosis and CRSwNP, we utilized the “VennDiagram” package in R software to overlap differentially expressed genes (DEGs), genes acquired through WGCNA, and genes present in ferroptosis gene sets. Histogram was used to illustrate the disparities in the expression patterns of core gene observed in both CRSwNP and CRSsNP sample. To perform the hypothesis tests, we employed 2 statistical methods: the *t*-test and the Mann–Whitney *U*-test. The *t*-test was utilized when the data followed a normal distribution, while the Mann–Whitney *U*-test was applied when this assumption was not met. For determining significance, a threshold of *P* < .05 was employed.

### 2.7. Enrichment analysis

In order to study the biological mechanism of hub genes affecting CRS, a functional enrichment analysis was performed. We first analyzed the Gene Ontology (GO) annotation and Kyoto Encyclopedia of Genes and Genomes (KEGG) pathway enrichment analysis involved in DEG and WGCNA intersection genes and used the “GOplot” package in R software, resulting in a chord plot that presented the conclusive findings. In the subsequent step, GSEA uncovered the respective functions of the genes derived from DEG, WGCNA, and the intersection genes within the ferroptosis gene set. According to the hub gene’s median expression level, the samples were categorized into 2 groups: a group with low-expression and another with high expression. The logFC sorted the genes in descending order. The “enrichplot” package in R software was employed to present the ultimate outcomes. Utilizing the “clusterProfiler” package in R software, all the analyses were performed with a filtering criterion of *P*_adj < .05.

### 2.8. Logistic regression model

Logistic regression, a generalized linear regression model, provides automated disease diagnosis. In this research, logistic regression is employed with dual response variables: 1 for CRSwNP sample and 0 for CRSsNP sample. To simplify the model, stepwise regression analysis is utilized to eliminate insignificant factors while retaining the significant ones. The model iteratively adds or removes variables until minimizing the Akaike Information Criterion (AIC). Subsequently, logistic regression is applied to establish the relationship between the significant factors and the response variable. To evaluate the model’s diagnostic efficacy, the receiver operating characteristic curve (ROC) is used, and the area under the ROC curve is calculated. These analyses were conducted using R software’s “stats” and “pROC” packages.

### 2.9. Immune infiltration and immune-related factors

The assessment of immune cell infiltration in the microenvironment was conducted through the utilization of CIBERSORT, a comprehensive tool comprising 547 biomarkers and 22 distinct types of human immune cells, encompassing plasma, B cells, T cells, and subsets of myeloid cells. The underlying principle of this tool rests upon the utilization of linear support vector regression, which engenders deconvolution analysis on the matrix expressing immune cell activity. In this study, the expression data for GSE36830 were employed to quantify the relative proportions pertaining to the 22 different immune cell categories present within each sample. Furthermore, Spearman correlation analysis was performed to evaluate the relationship between hub genes, immune infiltration, and immune factors. This analysis was executed employing the “psych” package within the R software, and the outcomes are visually depicted in the form of heat maps.

### 2.10. Drugs from the DrugBank

Search the DrugBank database for drugs that focus on the central gene. The DrugBank database serves as a comprehensive chemoinformatics and bioinformatics repository that provides an extensive amount of information regarding drugs and their targets. The database contains a wide range of more than 7800 medications, comprising different categories including nutraceuticals, investigational drugs, small molecule drugs approved by the FDA, and biotechnology drugs approved by the FDA.^[20]^ Moreover, DrugBank gathers a vast inventory of pharmacogenomics research-focused drugs that are associated with Single Nucleotide Polymorphisms (SNPs).

### 2.11. Quantitative PCR analysis

Total RNA was extracted using the RNeasy Plus Mini Kit (Qiagen, Hilden, North Rhine-Westphalia, Germany, 74,136). Reverse transcription was conducted with 100 ng of total RNA using the PrimeScript RT Reagent Kit (Takara, Kyoto, Shimogyo-ku, Japan, RR037A). The SYBR Green detection system (Applied Biosystems, Carlsbad, California, 4,367,659) and specific primer ALOX15 (also from Qiagen) were employed. Normalization was carried out using the housekeeping gene Gapdh, and the amount of mRNA in each experimental group was normalized relative to the control group. Primers used for RT-qPCR are listed in supplement qPCR.

### 2.12. Statistical analysis

All analyses were performed in R software. Choose *t*-test and Mann–Whitney *U*-test depending on the conformity of the data to the normal distribution. Typically, significance is defined by *P* < .05.

## 3. Results

### 3.1. Identification of hub genes associated with CRSwNP and ferroptosis

**Figure 2.**
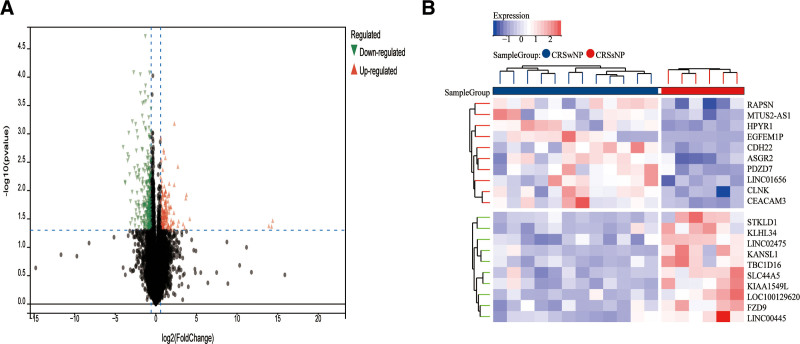
Differentially expressed genes between CRSwNP and CRSsNP samples. (A) Red genes represent significantly high expression in CRSwNP, green genes represent significantly high expression in CRSsNP, and gray genes indicate insignificant changes. (B) The heatmap shows the top 20 genes significantly highly expressed in CRSwNP or CRSsNP samples. CRSsNP = chronic rhinosinusitis without nasal polyps, CRSwNP = chronic rhinosinusitis with nasal polyps.

**Figure 3.**
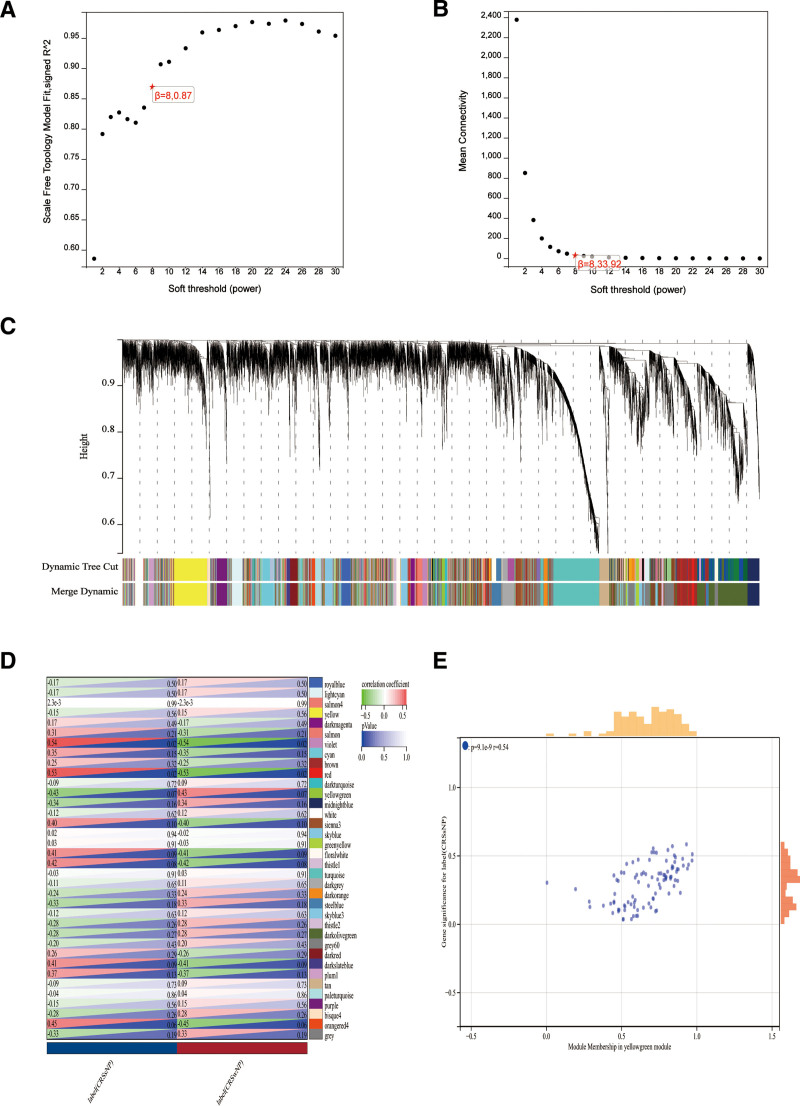
Results of the WGCNA. (A) The corresponding scale-free topological model fit indices at different soft threshold powers. (B) The corresponding mean connectivity values at different soft threshold powers. (C) Cluster dendrogram of genes. (D) Correlations between different modules and clinical traits. Red represents a positive correlation, and green represents a negative correlation. (E) Correlation of module membership and gene significance in the yellowgreen module. WGCNA = weighted gene co-expression network analysis.

**Figure 4.**
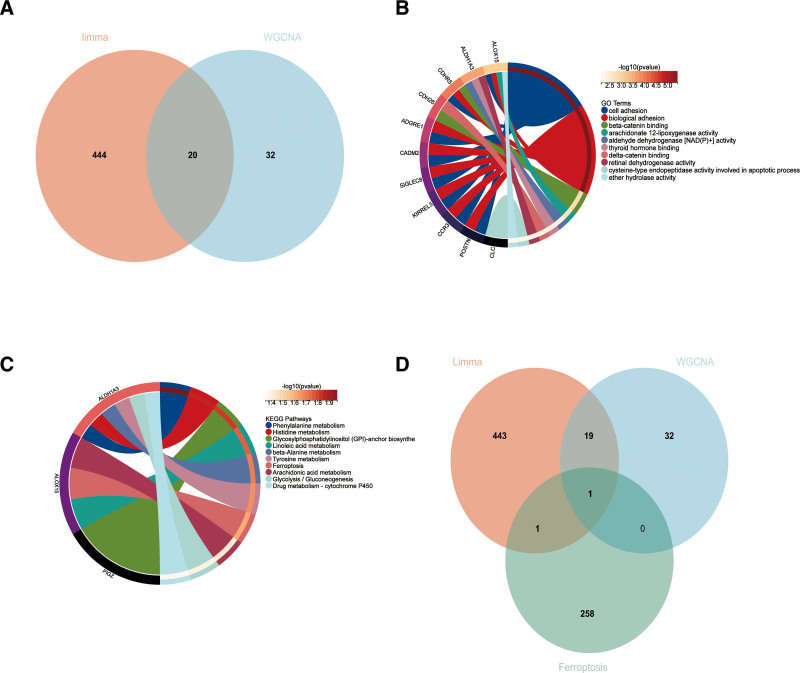
Hub genes, GO analysis and KEGG analysis. (A) One hub gene were obtained by taking the intersections of the DEGs, MEyellowgreen module genes of the WGCNA, and ferroptosis-related genes. (B) Biological processes in which the hub genes of CRS were involved. CRS = chronic rhinosinusitis, DEG = differentially expressed genes, GO = gene ontology, KEGG = Kyoto Encyclopedia of Genes and Genomes, WGCNA = weighted gene co-expression network analysis.

**Figure 5.**
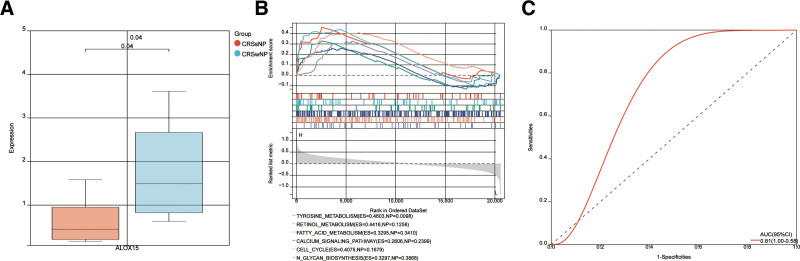
(A) Expression of ALOX15 in the CRSwNP and CRSsNP groups of superior turbinates samples GSE36830. (B) GSEA revealed the enriched pathways of ALOX15. (C) ROC curve of ALOX15 in CRSwNP and CRSsNP. ROC curve was drawn to evaluate the accuracy of ALOX15 in diagnosing CRSwNP. CRSsNP = chronic rhinosinusitis without nasal polyps, CRSwNP = chronic rhinosinusitis with nasal polyps, GSEA = gene set enrichment analysis, ROC = receiver operating characteristic curve.

In order to identify genes, associated with CRS, our initial step involved acquiring 464 genes that demonstrated differential expression from the dataset GSE36830. We applied a filtering criteria of “*P*_adj < .05 and abs(logFC) > .585” to obtain these DEGs. Visual representations of these DEGs were depicted through volcano plots (as shown in Fig. 2A). To further understand the data, a heatmap illustrating the top 20 differentially expressed genes was generated (refer to Fig. 2B). Subsequent to eliminating any abnormal samples and filtering out genes, we extracted the expression profiles of 20,549 genes and 18 samples from GSE36830. These profiles were then utilized to construct a gene co-expression network with a consideration for weights. By setting the soft threshold power to 8, we were able to achieve a scale independence value of 0.870 as well as an average connection value of 33.920 (as displayed in Fig. 3A and B). Through dynamic tree cutting, a total of 36 distinct co-expression modules were obtained when the minimum module size was set to 30 and the cutting height was 0.25 (Fig. 3C). Next, we conducted correlation analysis to examine the association between each module and clinical characteristics. Our findings revealed a significant positive correlation between the Violet module and CRSsNP (*R* = 0.44, *P* = 1.9e−6), and a positive correlation between the Yellowgreen module and CRSwNP (*r* = −0.54, *P* = 9.1e−9; Fig. 3D).We utilized the module feature vectors and gene expression to derive the correlation calculation method, resulting in obtaining MM. Through the application of the cutting standard (|MM| > 0.8), a total of 53 highly connected genes within clinically significant modules were singled out and chosen for subsequent analysis. Furthermore,the analysis of correlation between MM and GS indicated a strong association of these genes with both modules and phenotypes (cor = 0.61, *P* = 1.8e−161; Fig. 3E). 53 Violet, Yellowgreen module genes and 464 DE were intersected to obtain 20 CRS genes (Fig. 4A) for functional enrichment. 464 DE, 53 Violet, Yellowgreen module genes, and From the intersection of 520 ferroptosis-related genes (Table S1, Supplemental Digital Content, http://links.lww.com/MD/O2, central gene related to ferroptosis and CRS were obtained (Fig. 4D). The histogram showed that ALOX15 was highly expressed in the CRSwNP group (Fig. 5A).

### 3.2. Biological processes and pathways enriched for the hub genes

To understand the potential biological roles of these genes, enrichment analysis was performed. GO analysis showed that 20 genes were involved in cell adhesion, biological adhesion, aldehyde dehydrogenase [NAD(P)+] activity, etc (Fig. 4B). KEGG analysis showed that 20 genes were involved in glycolysis, gluconeogenesis, Ferroptosis, etc (Fig. 4C). GSEA results show that ALOX15 may affect multiple KEGG pathways, such as fatty acid, tyrosine, and retinol metabolism (Fig. 5B).

### 3.3. Construction a diagnostic model

**Figure 6.**
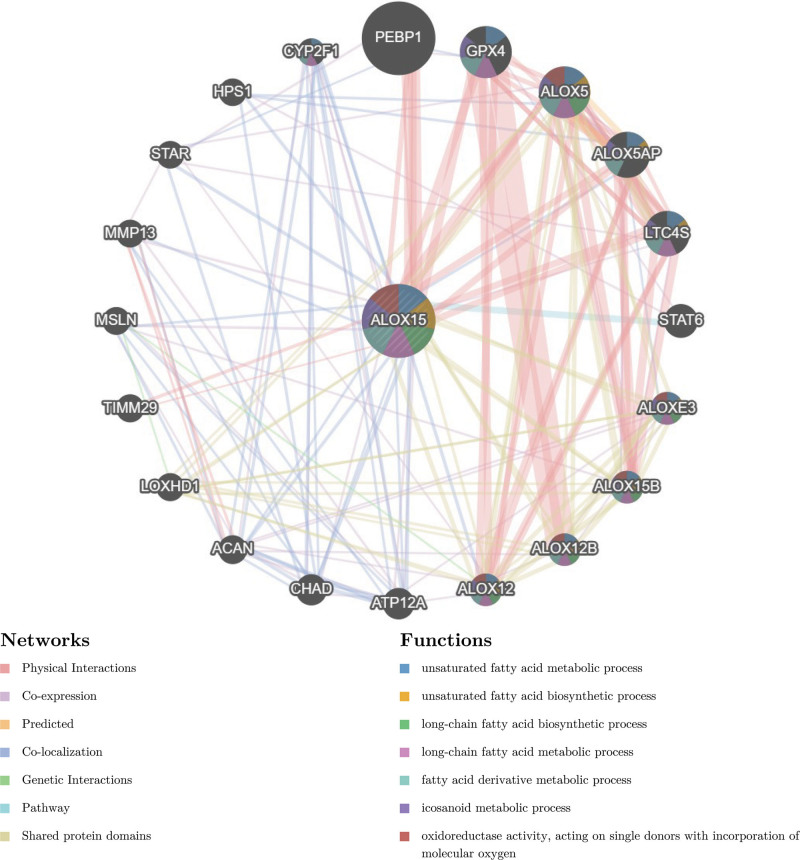
The gene–gene interaction network for ALOX15 was analyzed using the GeneMANIA database. The 20 most frequently changed neighboring genes are shown. The predicted genes are located in the outer circle, and ALOX15 are in the inner circle.

Based on GSE36830, a logistic regression algorithm was utilized to construct a multi-gene prediction model. Model was obtained through stepwise regression analysis with ALOX15. The diagnostic performance of the prediction model derived from the ALOX15 gene exhibited favorable result with an AUC of 0.82 (Fig. 5C). GeneMANIA performed PPI analysis on the ALOX15 gene, as well as their 20 interacting genes. The analysis aimed to predict co-localization, shared protein domains, co-expression, pathway predictions, and correlations (Fig. 6). The genes predicted can be found in the outer circle, whereas the ALOX15 gene can be found in the inner circle. As depicted in Figure 6B, the network demonstrates the enrichment of these genes in processes related to unsaturated fatty acids, including metabolic processes, biosynthetic processes, and oxidoreductase activity. These processes encompass unsaturated fatty acid metabolic process, unsaturated fatty acid biosynthetic process, long-chain fatty acid biosynthetic process, long-chain fatty acid metabolic process, fatty acid derivative metabolic process icosanoid metabolic process and oxidoreductase activity, acting on single donors with incorporation of molecular oxygen.

### 3.4. Immune infiltration

**Figure 7.**
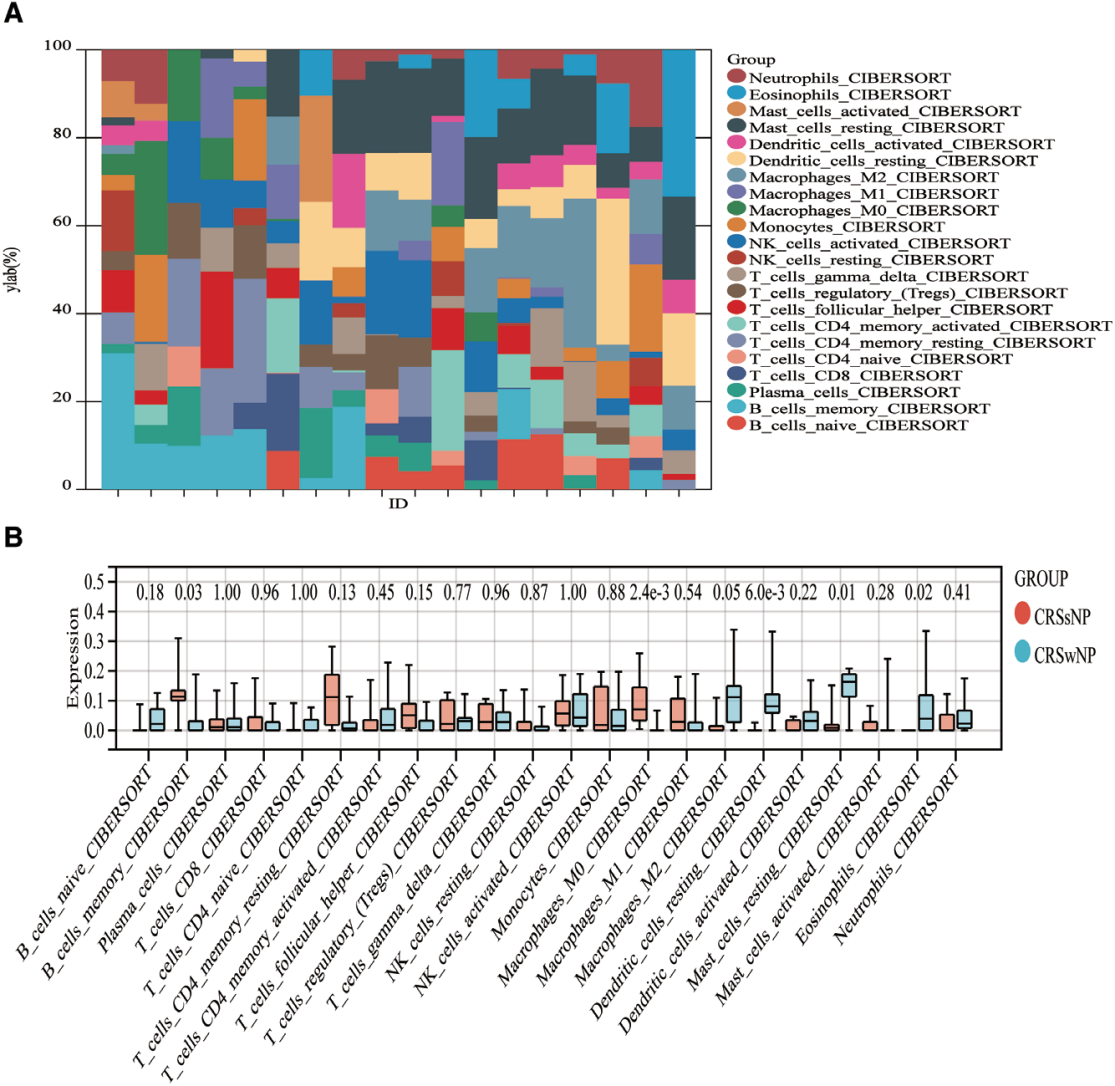
Immune infiltration between CRSwNP and CRSsNP samples. (A) The relative percentage of 22 immune cells in each sample. (B) Differences in immune infiltration between CRSwNP and CRSsNP samples. CRSsNP = chronic rhinosinusitis without nasal polyps, CRSwNP = chronic rhinosinusitis with nasal polyps.

**Figure 8.**
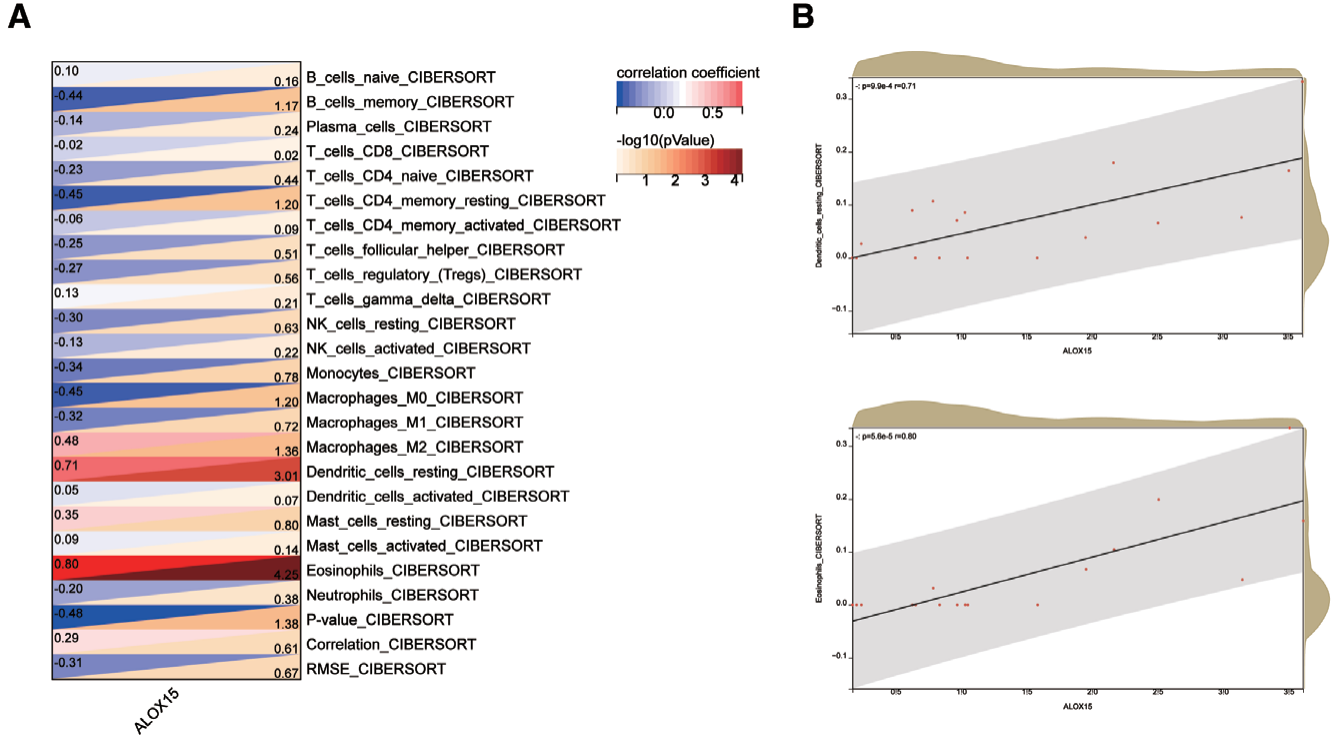
Correlation of ALOX15 with immune cells. (A) Immune cells. (B) Eosinophils and Dendritic_cells_resting.

The sensitivity of clinical treatment and disease diagnosis are greatly influenced by the microenvironment composed of immune cells, extracellular matrix, inflammatory factors, and various growth factors. The CIBERSORT algorithm was utilized in this investigation to estimate the proportion of 22 immune cells in a total of 18 nasal polyp samples, comprising 12 samples of CRSwNP and 6 samples of CRSsNP. The resulting histograms present a visual representation of these cell proportions (Fig. 7A). Comparisons in boxplots were conducted to evaluate the infiltration of immune cells in CRSwNP and CRSsNP samples (Fig. 7B). The results showed that naive B cells (*P* = .18), activated CD4 memory T cells (*P* = .45), activated NK cells (*P* = 1.00), M2 macrophages (*P* = .56), and activated trees in the CRSwNP group Proportions of neurite cells (*P* = .22), resting mast cells (*P* = .01), and eosinophils (*P* = .02) were significantly higher than resting CD4 memory T cells (*P* = .13), monocytes The proportions of cells (*P* = .88), M0 macrophages (*P* = 2.4e-3) and M1 macrophages (*P* = .54) were lower than those in the CRSsNP group. Subsequently, an analysis was performed to examine the association between hub genes and immune infiltration (Fig. 8A). ALOX15 was significantly positively correlated with Eosinophils and Dendritic_cells_resting (Fig. 8B).

### 3.5. Drugs from the DrugBank

**Figure 9.**
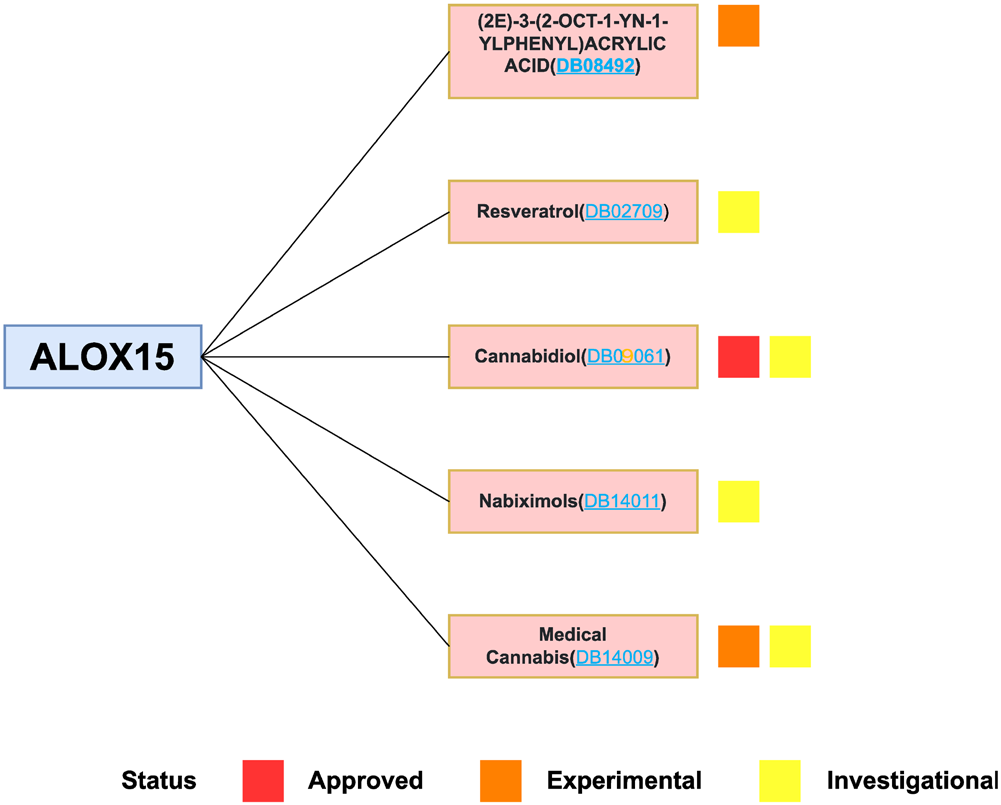
Drugs targeting ALOX15 obtained from the DrugBank database. Drug statuses, including approved, experimental, investigational, are indicated by colored squares.

Based on the drug and target information in the DrugBank database, 5 drugs targeting this central gene were identified (Fig. 9). Among these drugs, 1 drug is approved, 3 are investigational drugs, and 1 is an experimental drug. Cannabidiol (DB09061) is an active cannabinoid used as an adjunctive therapy for the treatment of epileptic seizures associated with Lennox–Gastaut syndrome or Dravet syndrome. It is also utilized for the alleviation of moderate to severe neuropathic pain or other painful conditions symptoms. Resveratrol (DB02709) is a phytoalexin that has been found to inhibit herpes simplex virus types 1 and 2 replication in a dose-dependent, reversible manner. The benefits of this substance encompass its anti-inflammatory and antioxidant properties. In preclinical studies, resveratrol was found to have potential anticancer properties. Medical Cannabis (DB14009) The main psychoactive component of cannabis, delta-9-tetrahydrocannabinol via cannabinoid-1 and cannabinoid-2, Nabiximols (DB14011) are derived from the cannabis species Cannabis sativa L. A whole plant extract that has been purified into the active ingredients cannabidiol and delta-9-tetrahydrocannabinol.

### 3.6. Experimental validation

**Figure 10.**
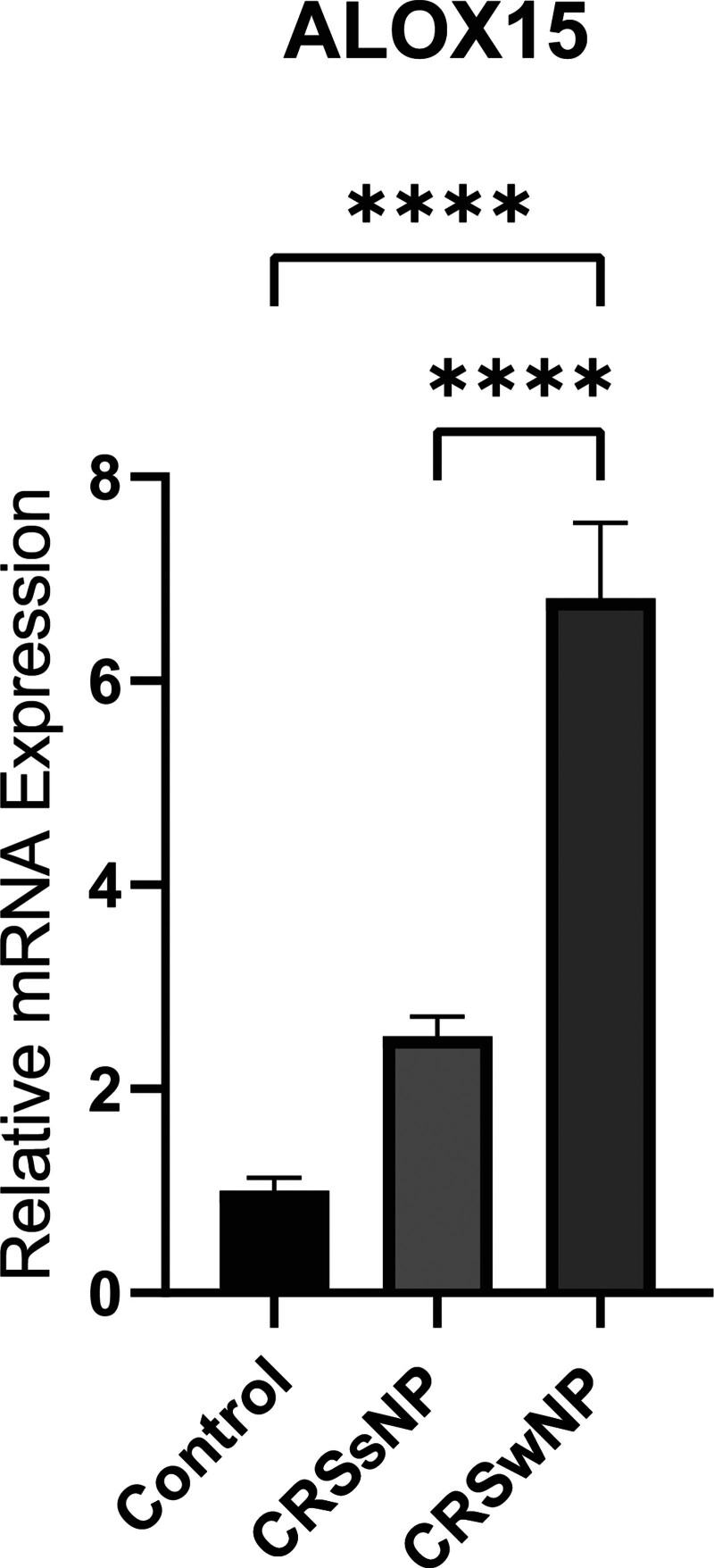
The expression levels of ALOX15 mRNA were significantly higher in patients with chronic rhinosinusitis with nasal polyps (CRSwNP) compared to control subjects. CRSwNP = chronic rhinosinusitis with nasal polyps.

Relative gene expression levels of ALOX15 were assessed by RT-qPCR using NP tissues from patients with CRSwNP and nasal mucosal samples from healthy controls. The expression level of ALOX15 was found to be significantly higher in CRSwNP patients compared to control subjects (*P* < .05, Fig. 10) (Table S2, Supplemental Digital Content, http://links.lww.com/MD/O3).

## 4. Discussion

Ferroptosis is a specific form of cell death associated with the accumulation of intracellular iron ions and lipid peroxidation. Ferroptosis is classified as regulated necrosis and is more immunogenic than apoptosis. Encouraging evidence suggests that ferroptosis plays a crucial role in inflammation.^[21–23]^ In addition, as an inhibitor of ferroptosis, it has demonstrated anti-inflammatory properties in various experimental models of specific illnesses.^[24,25]^ Therefore, it is important to study CRSwNP with The relationship between ferroptosis, further explaining its mechanism, and exploring more precise targeted therapy with ferroptosis inhibitors will be more promising research. In this study, we performed a comprehensive bioinformatics analysis. First, the hub gene related to ferroptosis and CRS including ALOX15 was obtained through differential analysis and WGCNA in GSE36830 nasal tissue samples. ALOX15 (arachidonate 15-lipoxygenase) is a protein-coding gene that produces an enzyme in cells called 15-lipoxygenase. This enzyme catalyzes the oxidation of fatty acids within cells, thereby producing a series of lipid peroxidation products. Pathways related to ALOX15 include fatty acid metabolism and tyrosine metabolism. As a ferroptosis-related marker, the upregulation of its expression occurs during erastin or RSL3-induced ferroptosis.^[26]^ Combining bioinformatics analysis and in vitro experiments, a recent study confirmed that ALOX15 is a ferroptosis-related gene and can accurately predict LUAD. Prognosis.^[27]^ In terms of ALOX15-related pathways, Jaewang Lee et al pointed out that increasing the expression of ALOX15 promotes polyunsaturated fatty acid peroxidation.^[28]^ Jie Zhao et al used the ALOX15 inhibitor PD146176 to partially reverse the ferroptosis in TBH-treated neurons induced by SSAT1 upregulation.^[29]^ Xiao-Hui Ma et al results indicate that ALOX15 induction during ischemia is the “burning point” that ignites phospholipid oxidation to ferroptosis signaling.^[30]^ A recent study showed that the upregulation of ALOX15 expression induces an increase in arachidonic acid, ROS accumulation and lipid peroxidation, ultimately causing ferroptosis.^[31]^ Hind Bouchaoui et al prevent ferroptosis by inhibiting ALOX15.^[32]^ In addition, Ragnar P Kristjansson et al demonstrated that ALOX15 can promote eotaxin-3 in NP of CRSwNP patients.^[33]^ However, it has not been explored in published studies of CRS or CRSwNP, and ALOX15 may play an important role in ferroptosis in CRSwNP. Using stepwise regression and logistic regression analysis, we employed the ALOX15 gene to develop a diagnostic model for CRS. The diagnostic performance of the model was found to be satisfactory, as evidenced by the high AUC value. Additionally, here, immune cell infiltration was further explored. The boxplot presents notable dissimilarities in different immune cells among the CRSwNP and CRSsNP groups. Previous studies have demonstrated the increased presence of eosinophils in individuals with CRSwNP. When an allergen binds to a specific IgE antibody, an allergen-IgE complex is formed. These complexes bind to IgE receptors on the surface of eosinophils and activate eosinophils. Secondly, inflammatory mediators such as histamine and leukotrienes can also directly activate eosinophils. Activated eosinophils release active substances in eosinophilic granules, such as histamine, leukotrienes, platelet-activating factor, etc, which can cause vasodilation, increased vascular permeability, and infiltration of inflammatory cells. In addition, mast cells and M2 macrophages were also upregulated in CRSwNP. Takabayashi et al found that the number of MCs of different phenotypes in CRS patients was higher than that in the control group, while the increase in the number of MCs in CRSwNP patients was more obvious, confirming that MCs are significantly associated with the occurrence of nasal polyps.^[34]^ M2 macrophages can participate in the CRSwNP tissue remodeling process by affecting the production of TGF-β and interacting with it. There is an abnormal increase in TGF-β expression in CRSwNP, which is affected by many factors, and M2 macrophages are 1 of the extremely important factors.^[35]^ The results indicate that the hub gene could potentially exert a significant influence on the immune response in CRS. In addition, 5 medications focusing on this pivotal gene were sourced from the DrugBank database, a few of which have demonstrated efficacy in addressing CRS or associated comorbid conditions. Cannabidiol (DB09061) is an active cannabinoid used as an adjunctive therapy in the treatment of epileptic seizures associated with Lennox–Gastaut syndrome or Dravet syndrome, and to relieve symptoms of moderate to severe neuropathic pain or other painful conditions. Cannabidiol (CBD) nanoemulsion preparation has significant therapeutic effect on chronic rhinitis, acute rhinitis, and sinusitis. Resveratrol (DB02709) is a phytoalexin. Other notable advantages of resveratrol encompass its anti-inflammatory and antioxidant effects. In preclinical studies, resveratrol was found to have potential anticancer properties. SW Kim et al concluded that resveratrol may help prevent eosinophilic CRSwNP and that a key mechanism of its action is thought to be its anti-inflammatory effects, particularly on eosinophils, through inhibition of lip oxygenation enzymatic pathway.^[36]^ Cho et al found that resveratrol can also improve CF-related rhinosinusitis.^[37]^

## 5. Conclusion

Through bioinformatics methods, we obtained hub gene related to ferroptosis and CRS. Exploring the biological processes and pathways they are involved in may reveal the relationship between ferroptosis and CRSwNP. Furthermore, the hub gene exhibited a correlation with diverse immune cells, suggesting that the ALOX5 gene might also have a significant impact on the immune microenvironment. ALOX15 is considered an important ferroptosis-related gene in CRSwNP. Additional experimental validation is required to validate these functions.

## Acknowledgments

We would like to thank all the authors who made outstanding contributions to this study.

## Author contributions

**Conceptualization:** Qian Guo.

**Data curation:** Qian Guo, Xinjie Qiao.

**Formal analysis:** Qian Guo, Xinjie Qiao.

**Methodology:** Qian Guo.

**Project administration:** Qian Guo.

**Resources:** Qian Guo, Shuman Huang.

**Software:** Qian Guo.

**Supervision:** Qian Guo.

**Validation:** Qian Guo.

**Visualization:** Qian Guo, Shuman Huang.

**Writing – original draft:** Qian Guo.

**Writing – review & editing:** Qian Guo, Dong Dong, Yulin Zhao.

## Supplementary Material


