# Supplementary material for: Hub genes, diagnostic model, and predicted drugs related to ferroptosis in chronic rhinosinusitis with nasal polyps
Source: Medicine (Baltimore). 2024 Nov 29;103(48):e40624. doi: 10.1097/MD.0000000000040624 (PMC11608670; doi:10.1097/MD.0000000000040624)
Supplement: Supplementary file 1 [file medi-103-e40624-s001.docx]

**郑州大学生命科学伦理审查委员会**

伦 理 审 查 报 告

| **项目名称：**金葡菌生物膜α毒素调控 NF-kB/TGF-β1/Smad 通路影响慢性鼻窦 炎黏膜重塑的机制研究 |
| --- |
| **项目负责人：** 赵玉林 |
| **伦理审查意见：**  经郑州大学生命科学伦理审查委员会审查，该项目研究内容 和过程遵循国际及国家颁布的有关生物医学研究的伦理要求，同 意该项目申请 2020 年度国家自然科学基金项目。  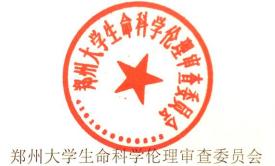  2020 年 4 月 16 日 |
